# U.S. Filipino Adults’ Patterns of CAM Use and Medical Pluralism: Secondary Analysis of 2012 National Health Interview Survey

**DOI:** 10.31372/20180303.1003

**Published:** 2018

**Authors:** Rhea Faye Felicilda-Reynaldo, Soyung Choi

**Affiliations:** a University of Hawaii at Manoa, Honolulu, Hawaii, USA; b University of Hawaii, John A. Burns School of Medicine, Honolulu, Hawaii, USA

**Keywords:** Asian American, complementary and alternative therapies, conventional medicine, Filipino, health behaviors, medical pluralism, secondary analysis

## Abstract

The cultural health beliefs of the Filipino population and colonial history of medicine in the Philippines could mean high use of complementary and alternative therapies (CAM) and increased engagement in medical pluralism (i.e., combined use of conventional medicine and CAM) among the U.S. Filipino population, the fourth largest immigrant group in the United States. However, there is limited research regarding U.S. Filipinos’ health behaviors related to CAM use and medical pluralism engagement. The purpose of this study was to explore patterns of CAM use and medical pluralism practices of Filipino adults living in the United States. Data from Filipino adult respondents of the 2012 National Health Interview Survey adult CAM supplement were analyzed to determine most common CAM used, most common conditions for which CAM was used as a treatment, reasons for using CAM for treatment of health conditions, and sources of recommendations for CAM as a treatment in this population. Dietary supplements were the most common CAM used by Filipino adults living in the United States. A small number of U.S. Filipino adults reported using CAM to treat pain-related and cardiovascular conditions, with the most common source of recommendations coming from friends and family members. Most common reasons for using CAM for treatment of health condition were: CAM is natural; CAM had a holistic approach, and CAM could be taken/practiced independently. Based on the findings of the study, Filipino adults living in the United States engage in a pluralistic approach to health by using CAM for treatment of health conditions. Analysis of the 2012 NHIS adult CAM supplement provided an overview of Filipino adults’ patterns of CAM use and medical pluralism; however, future research is still needed to explain such health behavior patterns.

Around one-third of the U.S. population has used a complementary and/or alternative therapy (CAM) (Clarke et al., 2015). The National Center for Complementary and Integrative Health (NCCIH) defines complementary and alternative therapies as “healthcare approaches developed outside of mainstream Western, or conventional, medicine” (2017, para. 3). Some CAM may require visits to practitioners; however, most CAM can be independently practiced or taken by individuals. Several studies have provided evidence that true use of alternative therapies (i.e., use of non-mainstream healthcare practices in place of conventional medicine) is rare, as health care consumers usually combine conventional and CAM therapies for promotion of health or treatment of illnesses (Davis et al., 2011; Malika et al., 2017; Rao, 2006; Wade et al., 2008). This combined use of conventional medicine and CAM is also known as medical pluralism (Wade et al., 2008).

The majority of research conducted on medical pluralism in developed countries (i.e., the United States and United Kingdom) has been done among minority populations, such as the Chinese (Green et al., 2006; Wade et al., 2007; Rochelle and Marks, 2010), Hispanic/Latino (Belliard and Ramírez-Johnson, 2005; Malika et al., 2017; Martinez, 2009; Sandberg et al., 2017), and Asian-Indian populations (De Gagne et al., 2015; Gupta, 2010; Rao, 2006; Rhodes et al., 2008). These three minority groups represent the three largest groups of immigrants to the United States (Migration Policy Institute, 2018) and are also known to use alternative health care systems, such as traditional Chinese medicine and Ayurveda, in their countries of origin; thus, research on CAM use and medical pluralism would be expected to focus on these populations. There is limited research on CAM use and medical pluralism among Filipinos, who are the fourth largest immigrant group in the United States (Migration Policy Institute, 2018).

## Filipinos: CAM Use and Medical Pluralism

An estimated 3.9 million Filipinos are living in the United States, making them the third largest Asian-American population in the United States (United States Census Bureau, 2017). This continuously growing Asian-American subgroup suffers significant health disparities. U.S. Filipinos have a high prevalence of cardiovascular disease (Ea, Colbert, Turk, & Dickson, 2018; Palaniappan et al., 2010) compared to other Asian-American subgroups. Cardiovascular disease is the leading cause of death in this population (Dalusung-Angosta et al., 2015). Filipinos are also vulnerable to diabetes and obesity. In a systematic review of studies on the prevalence of obesity and diabetes among Asian-Americans, Filipinos reported the highest mean for body mass index, had the highest proportion of people who were overweight, and had the highest prevalence of type 2 diabetes (Staimez et al., 2013). Filipinos also had the highest incidence and death rate of prostate cancer and the highest death rate of female breast cancer (McCracken et al., 2007). 

Despite the growing population and reports on health disparities, there is still limited research on Filipino health and health behaviors. The shortfall in science on Filipino health might be explained by two common research practices conducted in Asian-American health studies. Firstly, a large number of health studies on Asian-Americans have been done on individuals of East Asian descent (i.e., Chinese and Japanese) and then extrapolated on to the larger Asian-American group, which includes Filipinos. Secondly, health data collected from Filipinos have been grouped within the larger Asian-American category instead of being presented disaggregated (Holland & Palaniappan, 2012; Nadal et al., 2012). This research practice of treating multiple Asian-American subgroups as one homogeneous population is problematic as each of the more than 40 distinct Asian-American ethnicities distinct physical and cultural attributes including differing health beliefs, responses to illness, and health-seeking behaviors (Abesamis, Fruh, Hall, Lemley, & Zlomke, 2016).

Filipino history and culture play substantial roles in their health beliefs and practices. Due to the colonization of the Philippines by Spain and the United States, Filipinos have been exposed to Western medicine theories and practices (Joven, 2012). During the Spanish colonization period, medicines from Mexico and mainland Asia were imported to the Philippines. However, a lack of medical professionals meant that religious workers sent to the Philippines had to engage in healing roles, and a lack of medical supplies meant the use of local fauna which resulted in a mixed Western-herbal medicine practice (Joven, 2012). Additional health programs were developed when American armed forces helped propagate hygienic practices in Philippine *barrios* (neighborhoods) to reduce infectious diseases (Anderson, 2006). The mix of Western and herbal medicine is still actively practiced in the Philippines. The use of plants and herbs is a common health practice in the country; even the Philippine Department of Health has advocated the use of 10 different medicinal plants for general well-being and treatment of illnesses (Region 2 Veterans Regional Hospital, n.d.).

Aside from introducing Western medicine to the Philippines, the Spaniards and Americans also introduced different facets of the Christian religion to the Filipinos. Religion has always been woven into Filipino identity and culture; they have been known to manage difficulties and challenges in life by engaging in religious and spiritual practices such as attending church/mass, private praying, and religious processions (Gonzales, 2009). Multiple health studies have provided evidence of Filipinos’ predominant use of spirituality and prayer to cope with illness (Abe-Kim, Gong, & Takeuchi, 2004; Hsiao et al., 2006; Lagman et al., 2014).

Filipinos also have similarities to other Asian ethnicities, such as subscribing to the health principle of *timbang* (balance). The principle of *timbang* posits health develops due to balance and illness develops due to imbalance, brought about by humoral pathology and stress (Dela Cruz and Periyakoil, 2010). Due to their belief in the principle of *timbang*, Filipinos may choose to try using CAM to correct imbalances for holistic healing. Additional theories of illness subscribed to by Filipinos include mythical, personalistic, and naturalistic causes; these may encourage Filipinos to consult with folk medicine practitioners or shamans for the healing of illness they believe to be caused by mythical or naturalistic influences (Dela Cruz and Galang, 2008; Dela Cruz and Periyakoil, 2010; Hwang et al., 2007; Yamada et al., 1999).

A few studies have shown U.S. Filipinos engaging in the practice of medical pluralism. In a study comparing CAM use among breast cancer survivors of four ethnic/minority groups in Hawaii, Matsuno et al. (2012) reported Filipino Americans had higher utilization rates of alternative medical systems (Ayurveda, traditional Chinese medicine [TCM], homeopathic, or naturopathic therapies) compared to other subgroups including Japanese American, Native Hawaiian, and others. The researchers also discussed how participants in their study who had received surgery as a first-line treatment for breast cancer and had a comorbidity of diabetes were more likely to use CAM (Matsuno et al., 2012). In a qualitative study evaluating illness beliefs, perceptions, and practices of Filipino Americans regarding hypertension, some participants shared that, in conjunction with the use of pharmacological and nonpharmacological therapies prescribed through conventional medicine, they also used home remedies to treat their hypertension (Dela Cruz and Galang, 2008). These remedies included drinking herbal teas, using herbal poultices, and taking commercially available nonvitamin/nonmineral health supplements. Participants also reported using acupuncture as a complementary therapy, as well as receiving consultation from Filipino folk medicine practitioners (also known as *hilot*) to receive whole body massage to control their blood pressure (Dela Cruz and Galang, 2008).

The above-described historical, cultural, and health beliefs of Filipinos could mean high use of CAM among the U.S. Filipino population; however, not much is known about their CAM use. Medical pluralism engagement is also likely to be high among the U.S. Filipino population yet only limited research has been conducted on medical pluralism in this Asian-American subgroup; a subgroup which has been found to have higher rates of health insurance coverage than other immigrant subgroups and other foreign- and U.S.-born populations (Migration Policy Institute, 2018). Knowing U.S. Filipinos’ health behaviors related to CAM use and medical pluralism engagement could have significant implications to health care delivery to this population, including access (e.g., potential delay in care), safety (e.g., interactions between prescription drugs and natural herbs), and education (e.g., need address CAM use and medical pluralism when teaching patients about health/wellness and disease management). Also, if CAM use and medical pluralism have strong connections to the Filipino culture, these health behaviors should be included in culturally relevant health interventions for this population. Thus, more research needs to be conducted to better understand the U.S. Filipino’s health behaviors related to CAM use, as well as medical pluralism engagement in this population.

## Methods

### Study aim and research questions

Through a secondary analysis of the adult CAM supplement of the 2012 National Health Interview Survey (NHIS), we aimed to explore patterns of CAM use and medical pluralism practices of Filipino adults living in the United States.

Specifically, we analyzed the 2012 NHIS Adult CAM survey dataset to answer the following research questions:

1. What are the most common CAM therapies used by Filipino adults living in the United States?
2. Which types of health conditions do Filipino adults use their top three CAM therapies as treatment for?
3. What are the most common reasons why Filipino adults use their top three CAM therapies to treat a health condition?
4. Where do Filipino adults usually get their recommendations for their top three CAM therapies?

### Data source

The NHIS is a cross-sectional, face-to-face survey conducted by the National Center for Health Statistics (NCHS) and Centers for Disease Control and Prevention (CDC), and is the principal source of information regarding the health of the civilian, noninstitutionalized U.S. population (NCHS, 2013a). A statistically representative sample of the U.S. population is surveyed using Core and Supplemental questionnaires to gather data on demographics, socioeconomic characteristics, and a broad range of health information.

The four components of the core questionnaires include the household, family, sample child, and sample adult surveys. Additionally, the NHIS presents disaggregated health data on the three largest Asian-American subgroups: Chinese, Asian Indians, and Filipinos (NCHS, 2013a). Annually, different supplemental surveys are added to the core surveys to collect more data on additional health-related topics. Every 5 years, starting in 2002, data on CAM use among adult and child respondents has been collected. These supplemental surveys, also known as the Adult CAM and Child CAM supplements, are sponsored by the NCCIH (NCHS, n.d.), previously known as the National Center for Complementary and Alternative Medicine (NCCAM). Information regarding the development of the CAM supplement questionnaires is published elsewhere (Stussman et al., 2013). For this study, we used the 2012 Adult CAM Supplement to analyze patterns of CAM use and medical pluralism among Filipino adults living in the United States.

### Study population

The population of interest in this study is Filipino adults (18 years old and above) living in the United States. The Filipino race/ethnicity can be found under the variable code MRACRPI2 of the Sample Adult dataset of the 2012 NHIS (NCHS, 2013b). Although the NHIS survey collected data on CAM use among children, the researchers only chose to analyze data on adults as they have the autonomy to decide which healthcare approach(es) they would use for their health and well-being. The final analysis sample was 518 (unweighted count) Filipino adult respondents in the 2012 NHIS Sample Adult and Adult CAM surveys. Using the wtfa_sa weight of the 2012 NHIS survey for analysis of the Adult CAM data (NCHS, 2013a), the number of respondents translated to a weighted population estimate of 3,067,658 Filipino adults.

### 2012 NHIS variables

Table 1 summarizes the different variable codes used from the 2012 NHIS Adult CAM survey to conduct this study. This table summary includes any categorization, consolidation, and recoding of survey questions and variables in preparation for statistical analysis. Variable codes and labels are summarized in the document, *2012 NHIS Public Use Variable Summary: Adult Alternative Medicine* (NCHS, 2013b).

**Table 1:** Variables Used for Data Analysis; Includes Recategorization, Consolidations, and Recoding

Research Question	2012 NHIS Variables (Presented in Codes) Included in Analysis
1	COM_USE; MAS_USE; ACU_USE; EHT_USE; NAT_USE; HYP_USE;
	BIO_USE; AYU_USE; CHE_USE; CST_USE; TRD_EVR; AVT_USE;
	AHB_EVR; HOM_USE; MBO_USE; YTQE_YOG; YTQE_TAI; YTQE_QIQ;
	DITEVER1; DITEVER2; DITEVER3; DITEVER4; DITEVER5;
	MOVE_FLD; MOVE_ALX; MOVE_PIL; MOVE_TPI
Related to 2 to 4	Top three most important CAM therapies: ALT_TP31; ALT_TP32; ALT_TP33
2	Consolidated & Re-coded: CMBND_CND01 (Mental health conditions)
	Consolidated & Re-coded: CMBND_CND02 (Cardiovascular/circulatory system conditions)
	Consolidated & Re-coded: CMBND_CND03 (Other chronic conditions, other than Cardiovascular conditions)
	Consolidated & Re-coded: CMBND_CND04 (Pain-related conditions)
	Consolidated & Re-coded: CMBND_CND05 (Acute and other conditions)3Consolidated & Re-coded: CMBND_RS1 (Medical treatments were too expensive)
	Consolidated & Re-coded: CMBND_RS2 (Therapy combined with medical treatment would help)
	Consolidated & Re-coded: CMBND_RS3 (Medical treatments do not work for health problem)
	Consolidated & Re-coded: CMBND_RS4 (Medications cause side effects)
	Consolidated & Re-coded: CMBND_RS5 (Can be practiced/done on your own)
	Consolidated & Re-coded: CMBND_RS6 (It is natural)
	Consolidated & Re-coded: CMBND_RS7 (Focuses on whole person, mind, body, and spirit)
	Consolidated & Re-coded: CMBND_RS8 (Treats the cause, not just symptoms)
	Consolidated & Re-coded: CMBND_RS9 (Part of upbringing)4Consolidated & Re-coded: CMBND_REC1 (Medical doctor)
	Consolidated & Re-coded: CMBND_REC2 (Family member)
	Consolidated & Re-coded: CMBND_REC3 (Friend)
	Consolidated & Re-coded: CMBND_REC4 (Coworker)

Source: 2012 National Health Interview Survey Adult CAM survey.

Research question 1 provides an overview of the different CAM therapies ever used by U.S. Filipino adults. To determine the most common CAM therapies used by Filipino adults living in the United States, the survey questions used for analysis were “Used *[CAM therapy]* for health, ever.”

Research questions 2 to 4 are questions related to medical pluralism practices among U.S. Filipino adults. We explored one aspect of medical pluralism that has been addressed in the 2012 NHIS adult CAM supplement: the use of CAM for treatment of health conditions that are also treated in the conventional health care system. The 2012 NHIS survey questions related to CAM use for treatment of health condition, however, does not specify if the CAM therapies were used before, with, or after using the conventional health system to treat the health condition.

The survey questions in the 2012 NHIS adult CAM survey related to medical pluralism were focused on respondents’ top three most important CAM therapies. For these survey questions, respondents were asked to choose up to three CAM therapies which they considered to be their most important. Then, additional questions were asked related to these three CAM therapies, including the use of these CAM therapies for the treatment of health condition. Because this section in the 2012 NHIS adult CAM supplement does not encompass all types of CAM therapies, we also presented the most common [indicated by highest number of users] top three most important CAM therapies indicated by the U.S. Filipino adult population. This data came from variables ALT_TP31, ALT_TP32, and ALT_TP33.

To study which health conditions Filipino adults commonly treated with a CAM, the survey questions “Used *[top 1, top 2, top 3 CAM therapy]* for *[health condition]*” were analyzed. Respondents were queried on whether they used one of their top 3 CAM therapies for 88 conditions and symptoms (originally coded TP#_CND01 to 88; with the “#” indicating whether the CAM was indicated to be the top one, top two, or top three by the respondents). To reduce data for statistical analysis, we grouped similar conditions and symptoms to come up with five new variables: CMBND_CND1 to 5, ranging from mental health conditions to acute and other conditions. We then consolidated the responses for which Filipino adults indicated they used a CAM to treat a condition for the top three CAM therapies.

Table 2 provides a summary of the source variables for the recategorized variables of the different types of health conditions for which CAM was used as a treatment. Pain-related conditions were determined according to the different CAM effectiveness research related to pain. Acute conditions were categorized based on whether the health condition/symptoms stem from an injury or acute illness. Some health conditions/symptoms were categorized under “other” due to its potential to be categorized in either acute or chronic condition categories (e.g., abdominal pain and eczema/skin allergy).

**Table 2:** Recategorization of Variables RE: Health Conditions for Which CAM Was Used as a Treatment

Mental Health Conditions (CMBND_CND01)	Cardiovascular/Circulatory System Conditions (CMBND_CND02)	Other Chronic Conditions, other Than Cardiovascular (CMBND_CND03)	Pain-related Conditions (CMBND_CND04)	Acute and Other Conditions (CMBND_CND05)
Feeling anxious; worried Depression	High cholesterol Coronary heart disease Circulation problems (other than legs) Heart condition, other than coronary heart disease Hypertension Poor circulation in legs Any cardiovascular conditions	Cancer Asthma Lung/breathing problem Diabetes	Muscle/bone pain Arthritis Gout Joint pain/stiffness Chronic pain Nerve damage Back pain Severe headache/migraine	Abdominal pain Stomach/intestinal illnesses Memory loss/cognitive function loss Fracture/bone or joint injury Sprain/strain Fatigue/lack of energy more than 3 days Eczema/skin allergy Frequent stress Vision/seeing problems causing difficulty in activity Condition not elsewhere classified

Source: 2012 National Health Interview Survey Adult CAM survey.

To determine the most common reasons why Filipino adults living in the United States use CAM therapies, we consolidated the responses by Filipino adults for the top three CAM therapies in nine survey questions: “Used *[CAM therapy]*/saw *[CAM practitioner]* because *[reason]*.” The consolidated responses were recoded as CMBND_RS1 to 9. Lastly, to find out from whom Filipino adults mostly got their recommendations for CAM, we combined the responses of Filipino adults for the top three CAM therapies in four survey questions: “Used *[CAM therapy]*/saw *[CAM practitioner]* because it was recommended by *[type of person].*” The combined responses were recoded as CMBND_REC1 to 4. A biostatistician performed all response categorizations, consolidations, and variable recoding.

### Statistical analysis

Descriptive statistics, such as frequencies and cross-tabulations, were used to analyze patterns of CAM use and medical pluralism among Filipino adults living in the United States. All analyses were conducted using IBM SPSS Statistics software version 24 (IBM, 2016). Complex samples analysis techniques were used to account for the multistage, complex sample design of the NHIS (IBM, n.d.). Due to the small number of responses from Filipino respondents for the medical pluralism-related survey questions (sample size <30 for a response of “Yes” to use CAM for treatment of health conditions based on the five categories), we did not perform association/correlation analysis.

## Results

Table 3 provides a summary of the findings for research questions 1 to 4. Findings are presented in rank from highest to lowest. Participant responses with unweighted counts translating to population estimates of < 1% were not included in the Table 3.

**Table 3:** Summary of Research Findings

Research Question	Variables Labels/Names	% (SE)
Most common CAM therapies used by Filipino adults in the United States	Multivitamins/multiminerals	66.3 (2.7)
	Herbal/nonvitamin supplements	21.6 (2.3)
	Chiropractic/osteopathic manipulation	16 (2.2)
	Massage	15.4 (2.2)
	Yoga	10.4 (1.9)
	Acupuncture	4.1 (0.9)
	Pilates	3.9 (0.9)
	Meditation/guided imagery/progressive relaxation	3.0 (0.9)
	Vegetarian/vegan diet	2.4 (0.7)
	Taichi	1.3 (0.5)
	Traditional healers	1.3 (0.6)
	Homeopathy	1.2 (0.5)
Most common types of health conditions for which Filipino adults use CAM for treatment	Pain-related conditions	3.1 (0.9)
	Cardiovascular/circulatory system conditions	2.0 (0.8)
	Acute/other conditions	1.4 (0.5)
	Other chronic conditions, other than cardiovascular	1.2 (0.5)
Most common reasons Filipino adults use CAM to treat a health condition	It is natural	16.3 (1.9)
	Focuses on the whole person, mind, body, and spirit	14.7 (1.6)
	Can be practiced/done on your own	11.1 (1.8)
	Treats the cause, not just symptoms	10.2 (2.0)
	Part of upbringing	5.2 (1.4)
	Therapy combined with medical treatment would help	3.4 (0.8)
	Medical treatments do not work for health condition	1.6 (0.5)
Where do Filipino adults get their recommendations for CAM use	Friend	8.7 (1.7)
	Family member	8.6 (1.7)
	Medical doctor	5.0 (1.2)
	Coworker	2.1 (0.7)

The most common CAM therapies used by Filipino adults in the United States are multivitamin/multimineral supplements (66.3%), followed by herbal/nonvitamin supplements (21.6%). The third and fourth most commonly used CAM therapies by Filipino adults are chiropractic/osteopathic manipulation (16%), and massage (15.4%), respectively. The least commonly used CAM therapies were tai chi (1.3%), traditional healers (1.3%), and homeopathy (1.2%).

Table 4 provides a summary of the most common top three CAM therapies indicated by Filipino respondents of the 2012 NHIS adult CAM survey. The most common top one CAM treatment was a herbal therapy (taken from variable code: AHB_TP21). The most common top two and top three CAM therapies were practicing either yoga, tai chi, or qigong.

**Table 4:** Most Common Top Three CAM Therapies by U.S. Filipino Adults

First % (SE)	Second % (SE)	Third % (SE)
Herb 1 from AHB_TP21	Yoga/tai chi/qigong	Yoga/tai chi/qigong
10.7 (1.4)	2.4 (0.8)	1.8 (0.6)

Filipino adults in the United States most commonly used their top three CAM therapies for treatment of pain-related conditions (3.1%), such as arthritis and back pain. Filipino adults also used their top three CAM therapies to treat cardiovascular/circulatory system conditions (2.1%), which included hypertension, high cholesterol, and poor circulation in the legs. Acute/other conditions (1.4%) and other chronic conditions, not including cardiovascular (1.2%) were ranked third and fourth, respectively. Filipino adults engaged in medical pluralism (using their top three CAM therapies for treatment of health condition) because the CAM therapies are natural (16.3%), have a holistic approach (14.7%), and could be used or practiced on their own (11.1%). The least common reasons for using their top three CAM therapies for treatment of health condition were: using CAM therapy to treat the condition is part of their upbringing (5.2%), believing that CAM combined with medical treatment would help (3.4%), and indicating that medical treatments did not work for the condition they used a CAM for treatment (1.6%).

Lastly, Filipino adults usually received recommendations for their top three CAM therapies from their friends (8.7%) or family members (8.6%). The next highest source of recommendations for CAM therapies were medical doctors (5.0%). Filipino adults were least likely to get recommendations about CAM therapies from their coworkers (2.1%).

## Discussion

This is the first study, using secondary analysis of a national health survey, to assess CAM use and medical pluralism solely within the U.S. Filipino population. According to the 2012 NHIS data, Filipino adults living in the United States are likely to use dietary supplements (i.e., multivitamin/multimineral supplements and herbal products) for health purposes. These supplements are products intended for diet supplementation and are not considered drugs; thus, they are not intended for use to prevent, diagnose, and treat diseases (USFDA, 2017). It is not fully understood why U.S. Filipino adults take dietary supplements although it is possible that they use them to improve or maintain health, as found in the study by Bailey et al. (2013). Further research is needed to explain the high use of dietary supplements in this population.

It was interesting to note that only 1.3% of U.S. Filipino adults consulted a traditional healer. A study conducted by a collaboration of Asian and Filipino organizations in Los Angeles found 10.8% of Filipinos consulted a *hilot*, a traditional healer who provides massage therapy (The Historic Filipinotown Health Network & Semics LLC, 2007). Dela Cruz and Galang (2008) also found some participants received whole body massage therapy from *hilots* to treat their hypertension. Another earlier qualitative study presented information that Filipinos believed consulting an *albularyo*, another type of traditional healer, for herbal concoctions is an effective treatment for tuberculosis (Yamada et al., 1999). The low prevalence of use of traditional healers among U.S. Filipino adults from the 2012 NHIS CAM dataset may be attributed to unfamiliarity with the types of traditional healers respondents were queried about. Most of the traditional healers asked in the survey were of either from Native American or Hispanic/Latino cultures, such as the medicine man, *curandero, sobadores*, etc. U.S. Filipinos may have indicated their consultations with *hilots* as a type of massage therapy, which was the fourth most common CAM therapy they used.

Around 3% of Filipino adults used CAM as a treatment for pain-related conditions, such as back pain, arthritis, and migraines. Pain is a common reason for health care consumers to use complementary and alternative health approaches (NCCIH, n.d.). A systematic review of complementary approaches for self-management of chronic pain syndromes have found some evidence that yoga and tai chi may be helpful for chronic pain; however, the evidence is not strong enough to recommend use (Crawford et al., 2014). Filipino adult respondents of the 2012 NHIS have indicated their second and third most important therapies were either yoga, taichi, or qigong.

Another interesting finding from this secondary analysis was that 2% of Filipinos used CAM for treatment of cardiovascular/circulatory system conditions, such as high cholesterol and hypertension. Dela Cruz and Galang (2008) found Filipino Americans used home remedies, such as herbal teas, dietary supplements, and consultations with traditional healers as part of their management of hypertension. Further research should be conducted to understand this practice of including CAM therapies in the self-management of cardiovascular conditions.

U.S. Filipino adults valued the CAM therapies they used for treatment of health conditions which were natural, had a holistic approach, and could be independently practiced. The most important CAM therapy for Filipinos was herbal supplements. There is a widespread perception that herbal supplements are “natural” and thus safe to use (McCuistion, Vuljoin-DiMaggio, Yeager, and Winton, 2018). However, research has provided evidence that herbal products may interact with pharmaceutical products, potentially affecting pharmacokinetic and pharmacodynamic properties, which can result in adverse drug reactions such as toxicity (Bent, 2008; Izzo & Ernst, 2009). The interest in using CAM due to its holistic approach may be attributed to Filipinos’ beliefs on the principle of *timbang.* Lastly, Filipinos may be interested in CAM that can be practiced on your own as it will be low cost and under their control.

Around 5% of Filipino adults who used CAM for treatment of a health condition indicated CAM therapies were part of their upbringing. Due to the vague way this survey question was asked, it is not clear whether the term upbringing is related to the general Filipino cultural beliefs about health and healing or related to family practices. Additional research on the use of CAM as part of the upbringing of Filipinos should be conducted to elucidate this observation.

Filipino adults living in the United States received recommendations for CAM use for treatment from friends and family members, with an almost equal number of responses for the two categories. Filipinos are family-centric with family cohesiveness having a significant influence on one’s decision-making especially when related to health (Cura, 2015). Decision-making by Filipinos emphasizes the use of the concept of collectivism, wherein families make decisions together to think about the greater good of everyone involved (McLaughlin & Braun, 1998).

Although the family is the basic social unit in Filipino culture, kinship extends to those outside of family too. Friends may be treated as “one of the family” due to the relative flexibility of social relations within Filipino culture (Torres, 1985). Thus, recommendations by friends related to health care may be highly valued by Filipino adults living in the United States.

Five percent of Filipino adults living in the United States took recommendations from their medical doctor regarding CAM therapies. Filipinos’ respect for authority extends to medical personnel, and they may feel the need to be compliant with prescribed health therapies due to deference to their health care providers (McLaughlin and Braun, 1998). Future research should be conducted to determine whether it was the Filipino patient who asked for recommendations for CAM therapy or if their healthcare provider prescribed CAM as part of treatment for health conditions.

### Limitations

There are several limitations of this secondary analysis of the 2012 NHIS adult CAM survey. Firstly, the 2012 NHIS was only conducted in 32 states and the District of Columbia (NCHS, 2013a). Therefore, it is unclear whether the data gathered accurately represents the U.S. Filipino population at large; the findings of this analysis may not apply to Filipinos living in the 18 states who were not included in the survey. Conducting a nationwide survey among U.S. Filipinos, similar to the one done by Misra et al. (2010) on the Asian-Indian population, would provide a clearer picture of Filipinos’ health behaviors related to CAM use and medical pluralism. A nationwide study on Filipino health and health behaviors would also provide better evidence-based and culturally competent information to health care professionals, so they can effectively address this population’s health needs.

Secondly, the NHIS is a cross-sectional study; thus, the findings may only apply to a specific point in time. Trend analysis of CAM use and medical pluralism engagement among Filipino adults in the United States should be conducted in future research studies.

There is also a potential for selection bias in the survey. The NHIS does not collect data from hospitalized and institutionalized people; thus, CAM use and medical pluralism engagement among these populations cannot be determined through this survey (Falci et al., 2016). Also, frequency of CAM use was not a question asked in the survey. As stated by Falci et al., “if users do not use a modality frequently…[respondents] should be considered nonusers…” (2016, p. 4). Furthermore, we decided to use survey questions related to any CAM use ever (i.e., in the past or within 12 months of the survey); thus, findings from this analysis should not be interpreted to mean current CAM use and medical pluralism engagement.

Lastly, we only looked into one aspect of medical pluralism engagement—the use of CAM for treatment of health condition which could also be treated by the conventional health system—as provided by the 2012 NHIS adult CAM supplemental data. Based on the data, we are unable to determine if CAM therapies were used by the respondents before, with, or after the use of conventional health systems to treat the health condition. Thus, future research should also look into the patterns of schedule of use of CAM and conventional health system for treatment of a medical condition.

## Relevance to Nursing Practice and Conclusion

This study bridges the gap of research-based information on CAM-related health behaviors of U.S. Filipinos. We found U.S. Filipino adults engage in a pluralistic approach to health by using complementary and alternative approaches—including the use of CAM for treatment of different health conditions. However, more research needs to be done to further explain U.S. Filipino adults’ pattern of CAM and medical pluralism engagement, specifically in determining how CAM use affects individuals’ patterns of accessing conventional treatments. Additional research in this field will better inform nurses and other healthcare professionals regarding the impact of this group’s CAM-related behaviors on their health care needs. 

Nurses can use the findings of our study to enhance their knowledge of CAM-related behaviors of their Filipino patients. Our research will also help nurses become more aware of the concept of patient medical pluralism, which could have significant implications to health care access, patient safety, and patient education. 

## Declaration of Conflicting Interests

The author(s) declared no potential conflicts of interest concerning the research, authorship, or publication of this article.

## Funding

This work was partially supported by the National Institutes of Health (NIH) under grants U54MD007584 and U54MD007601 and by Sigma Theta Tau International Phi Gamma Chapter’s Research Funds. The content is solely the responsibility of the authors and does not necessarily represent official views of NIH.
